# Plasma trimethylamine n-oxide is associated with renal function in patients with heart failure with preserved ejection fraction

**DOI:** 10.1186/s12872-020-01669-w

**Published:** 2020-08-28

**Authors:** Fei Guo, Xueting Qiu, Zhirong Tan, Zhenyu Li, Dongsheng Ouyang

**Affiliations:** 1 Department of Clinical Pharmacology, Xiangya Hospital, Central South University, 87 Xiangya Road, Changsha, 410008 PR China; 2 Hunan Key Laboratory of Pharmacogenetics, Institute of Clinical Pharmacology, Central South University, Changsha, China; 3 Engineering Research Center of Applied Technology of Pharmacogenomics, Ministry of Education, Changsha, China; 4 National Clinical Research Center for Geriatric Disorders, Changsha, China; 5 Hunan Key Laboratory for Bioanalysis of Complex Matrix Samples, Changsha Duxact Biotech Co., Ltd., Changsha, China; 6 Department of Geriatric Medicine, Xiangya Hospital, Central South University, 87 Xiangya Road, Changsha, 410008 PR China

**Keywords:** HFpEF, TMAO, Renal dysfunction, eGFR, Biomarker

## Abstract

**Background:**

Heart failure with preserved ejection fraction (HFpEF) is an emerging global health problem with less awareness. Renal dysfunction in HFpEF is associated with worse outcome. However, there is lack of rapid, noninvasive and accurate method for risk stratification in HFpEF and renal dysfunction. This study aimed to explore the utility of plasma trimethylamine n-oxide (TMAO) for evaluation of HFpEF and renal function.

**Methods:**

Plasma TMAO levels were measured in total 324 subjects comprising 228 HFpEF patients and 96 healthy controls.

**Results:**

TMAO levels were significantly elevated in patients with HFpEF compared with controls (12.65(9.32–18.66) μg/l vs 10.85(6.35–15.58) μg/l, *p* <  0.01). Subjects in higher TMAO tertile group had more incidences of HFpEF ((78.5%) in tertile 3 vs (73.39%) in tertile 2 vs (59.26%) in tertile 1, *p* <  0.01). TMAO concentrations were inversely correlated with estimated glomerular filtration rate (eGFR) and HFpEF patients with impaired renal function (eGFR < 60 ml/min/1.73 m^2^) had higher TMAO than those with normal eGFR (≥ 60 ml/min/1.73 m^2^) (14.18(10.4–23.06) μg/l vs 10.9(7.48–15.47) μg/l, *p* < 0.01). Increased TMAO levels were independently associated with higher risk of HFpEF (OR = 3.49, 95% CI: 1.23–9.86, *p* = 0.02) and renal dysfunction (OR = 9.57, 95% CI: 2.11–43.34, *p* < 0.01) after adjustment for multiple traditional risk factors. Furthermore, TMAO had good performance at distinguishing HFpEF from controls (AUC = 0.63, *p* < 0.01), and renal dysfunction from normal renal function in HFpEF (AUC = 0.67, *p* < 0.01).

**Conclusion:**

In this cross-sectional study, HFpEF and renal function were closely related with plasma TMAO levels and TMAO may serve as a diagnostic biomarker for HFpEF and renal function.

## Background

The population affected by heart failure (HF) is growing rapidly nowadays in the world due to ageing and increased incidence of cardiovascular disease risk factors such as obesity and hypertension. Heart failure with preserved ejection fraction (HFpEF) is a diverse syndrome marked by myocyte hypertrophy, concentric left ventricular remodeling, and end-diastolic stiffness. Patients with HFpEF have clinical features of heart failure but exhibit normal or near-normal left ventricular ejection fraction (EF), usually 50% or above. HFpEF patients have a higher morbidity than those with reduced ejection fraction (HFrEF) [1], but current therapies which are beneficial for HFrEF have failed to improve the outcomes of HFpEF according to recent data [2]. A major drawback regarding the development of new therapies for HFpEF, is the absence of clear diagnostic criteria, which bring difficulties to the definition of patients in clinical diagnosis. At present, the diagnosis of HFpEF is solely based echocardiography, which is somehow low in resolution, and the results are also susceptible to physicians’ clinical skills.

HFpEF is commonly associated with several comorbidities such as obesity, anemia, diabetes mellitus, and renal dysfunction. Data showed that between 20 and 40% of patients with acute heart failure could eventually develop some degree of renal failure [3]. Notably, renal function impairment in HF patients might in turn accelerates vascular stiffness and portends high risk of hospitalization as well as cardiovascular and all-cause mortality. Study have reported 1.63 folds higher risk of all-cause mortality in HF patients with increased serum creatinine levels compared to those with normal serum creatinine [4]. Although, the treatment of kidney disease and HF improved over the last decades, it still could not decrease the overall all-cause and cardiovascular mortality in HFpEF. Thus, developing sensitive, noninvasive biomarkers for auxiliary diagnosis of HF and evaluation of renal function, which may lead to favorable clinical outcomes and avoid the risk of end-stage renal disease in HF and cardiovascular mortality, is of great clinical importance.

Recently, multiple studies have suggested the strong association of gut microbiota with pathogenesis and progression in cardiovascular disease [5, 6]. The gut microbiota is influenced by dietary intake and in turn produce metabolites that contribute to affect the metabolism and immunity of host. Remarkably, trimethylamine N-oxide (TMAO), a gut microbial metabolite, was indicated to be a proatherogenic factor. Study conducted by Hazen firstly reported elevated TMAO in both atherosclerosis patients and mice model in 2011 [6]. Afterwards, several studies indicated that elevated TMAO level could predict an increased risk of major adverse cardiovascular events [7–9]. Results from Leong and his colleagues’ study elucidated the associations between increased TMAO levels with adverse outcomes in HF patients [10]. There were evidences that also indicated the association of abnormal TMAO concentrations with renal dysfunction, patients with end-stage renal disease have significantly elevated plasma TMAO concentrations [11]. However, the correlations of plasma TMAO levels with HFpEF and impaired renal function in HFpEF has not been investigated, and whether TMAO could be utilized as a biomarker for evaluation of HFpEF and renal function in HFpEF is still unknown. In this cross-sectional study, we aimed to explored the relationship between plasma TMAO levels with HFpEF and renal dysfunction, defined as estimated glomerular filtration rate (eGFR) < 60 ml/min/1.73 m^2^, in a hospital-based southern Chinese cohort.

## Materials and methods

### Study population

We recruited 228 patients (HFpEF group) who were hospitalized for coronary angiography and echocardiography in Xiangya hospital at Central South University, with a diagnosis of HFpEF between June 2014 to September 2019. HFpEF was defined as documented diagnosis of heart failure with ejection fraction > 50% (35 ≤ age ≤ 70). Another 96 healthy controls (CON group) was an independently recruited set who either visited hospital for health screen with no history or symptoms of HF, or underwent coronary artery CT or echocardiography for chest pain but showed negative results. The HFpEF group were further divided into eGFR < 60 group (*n* = 152) and eGFR ≥60 group (*n* = 76) according to eGFR score that calculated with an equation of Modification of Diet in Renal Disease (MDRD) as previously described [12]: GFR (expressed in ml/min/1.73 m^2^) = 186 × [serum creatinine (mg/dl)] ^–1.154^ × (age) ^− 0.203^ × (0.742 if female).

We exclude subjects with an active infection, malignancy, severe liver or cerebrovascular diseases, moderate-to-severe valvular heart disease, uncontrolled hypertension, severe proteinuria (> 3.5 g/day), intestinal dysfunction, gastrointestinal surgery history, organ transplants, choline or betaine or l-carnitine supplementation in recent 6 months, and those who received probiotics or antibiotic treatment within 3 month of enrollment to minimize potential confusing factors.

General information including age, sex, weight and height was retrospectively collected from each subject’s medical records. All subjects were informed before enrolled in the study. The study plan was approved by the Ethical Committee of Xiangya Hospital of Central South University.

### Laboratory test

Blood samples were collected using vacutainer tubes after at least 12 h of fasting, then immediately centrifuged and stored at − 70 °C until analysis. Plasma TMAO were measured by high performance liquid chromatography-tandem mass spectrometry (HPLC-MS) using d9-(trimethyl)-labeled internal standards as described previously [11]. To be brief, in 20 μl of plasma, proteins were precipitated with methanol containing 1 μg/l d9-TMAO as an internal standard. After centrifugation an aliquot of the supernatant was diluted with acetonitrile and subsequently analyzed.

All laboratory analysis were conducted by automatic biochemical analyzer (HITACHI7170S). Body mass index (BMI) was calculated by weight (kg)/height^2^ (m^2^). Blood pressure was measured after resting for at least 15 min.

### Statistical analysis

Statistical presentation and analysis of the current study were performed using the computer SPSS program (Statistical Package for the Social Science, Chicago). Categorical variables are presented as numbers and percentages. Continuous data are presented as mean ± standard deviations (SD) for normal distribution parameters or median (interquartile ranges, IQR) for non-normal distribution parameters. Student’s *t-*test or the Mann-Whitney *U* test was used for differences evaluation between two groups. Comparison between the tertiles was performed using one-way ANOVA. The Spearman correlation analysis was used to examine the correlation between TMAO and eGFR. Logistic regression analysis was performed to examine the odds ratio (OR) and 95% confidence interval (95% CI) of TMAO in HFpEF and in HFpEF with renal dysfunction. Adjustments were made for variables including: age, gender, systolic blood pressure (SBP), percentage of glycosylated hemoglobin (HbA1c%), Creatinine (Cr), triglyceride (TG), high-density lipoprotein (HDL) and low-density lipoprotein (LDL). Because the distributions of TMAO were skewed, they were log-transformed in logistic analysis. The area under the receiver-operating characteristic curve (AUC) was calculated to evaluate the diagnostic value of TMAO in discriminating HFpEF from healthy controls as well as HFpEF with renal dysfunction from those without. A two-tailed *p* value < 0.05 was considered statistically significant.

## Results

### Patients characteristics

Of the 228 HFpEF patients and 96 healthy controls, the baseline patient characteristics are displayed in Table 1 as categorized by HFpEF and CON group. The mean age of HFpEF patients were older than healthy controls (62 ± 1 vs 56 ± 1, *p* < 0.01). 62.72% of HFpEF and 59.38% of CON groups were male. HFpEF patients exhibited higher SBP (135 ± 1 mmHg vs 127 ± 2 mmHg, *p* < 0.01), fasting glucose (Glu) (5.42(4.87–6.11) mmol/l vs 5(4.68–5.53) mmol/l, *p* < 0.01), HbA1c% (6.1(5.9–6.2) vs 5.7(5.5–5.7), *p* < 0.01), Cr (91.95(78.85–103.8) μmol/l vs 82.6(74.3–95.75) μmol/l, *p* < 0.01), TG (1.69(1.13–2.48) mmol/l vs 1.35(1–1.8) mmol/l, *p* < 0.01), LDL (2.72(2.22–3.49) mmol/l vs 2.55(2.04–3.01) mmol/l, *p* < 0.01) and white blood cell (WBC) (6.8(5.5–7.8) × 10^9^/l vs 6.1(5–7.53) × 10^9^/l, *p* < 0.01) levels than control group. While decreased HDL (1.04(0.87–1.22) mmol/l vs 1.12(0.94–1.43) mmol/l, *p* = 0.01) and eGFR (54.85(46.57–64.26) ml/min/1.73m^2^ vs 62.03(52.64–70.74) ml/min/1.73m^2^, *p* < 0.01) was observed in HFpEF patients compared with controls. We found no significant differences of height, weight, BMI, diastolic blood pressure (DBP), heart rate (HR), total cholesterol (TC), alanine aminotransferase (ALT), glutamic-pyruvic transaminase (AST), creatine kinase-MB (CK-MB) and percentage of ejection fraction (EF%) between different groups (*p* > 0.05) (Table 1).

**Table 1:** Baseline characteristics of subjects stratified by CON and HFpEF

	CON (*n* = 96)	HFpEF (*n* = 228)	*p*
Male (n, %)	57 (59.38%)	143 (62.72%)	0.6
Age (years)	56 ± 1	62 ± 1	< 0.01
Height (cm)	163 ± 1	162 ± 0	0.67
Weight (kg)	65 ± 1	66 ± 1	0.76
BMI (kg/m^2^)	24 ± 0	25 ± 0	0.57
SBP (mmHg)	127 ± 2	135 ± 1	< 0.01
DBP (mmHg)	76 ± 1	77 ± 1	0.14
HR (bpm)	71 ± 1	72 ± 1	0.71
Glu (mmol/l)	5 (4.68–5.53)	5.42 (4.87–6.11)	< 0.01
HbA1c (%)	5.7 (5.5–5.7)	6.1 (5.9–6.2)	< 0.01
Cr (μmol/l)	82.6 (74.3–95.75)	91.95 (78.85–103.8)	< 0.01
eGFR (ml/min/1.73m^2^)	62.03 (52.64–70.74)	54.85 (46.57–64.26)	< 0.01
TG (mmol/l)	1.35 (1–1.8)	1.69 (1.13–2.48)	< 0.01
TC (mmol/l)	4.27 (3.63–4.89)	4.38 (3.65–5.38)	0.09
HDL (mmol/l)	1.12 (0.94–1.43)	1.04 (0.87–1.22)	0.01
LDL (mmol/l)	2.55 (2.04–3.01)	2.72 (2.22–3.49)	< 0.01
ALT (U/l)	21.3 (16.3–31.68)	24.2 (15.7–36.73)	0.5
AST (U/l)	22 (19.11–26.65)	23.45 (18.98–30)	0.35
WBC (10^9^/l)	6.1 (5–7.53)	6.8 (5.5–7.8)	< 0.01
CK-MB (U/l)	16.45 (13.13–21.65)	15.3 (11.8–22.5)	0.3
EF (%)	61 (57–67)	59 (55–63)	0.35
TMAO (μg/l)	10.85 (6.35–15.58)	12.65 (9.32–18.66)	< 0.01

Continuous data are presented as mean ± SD or median (interquartile range), and categorical variables are presented as counts and percentage (%)

**Fig. 1:**
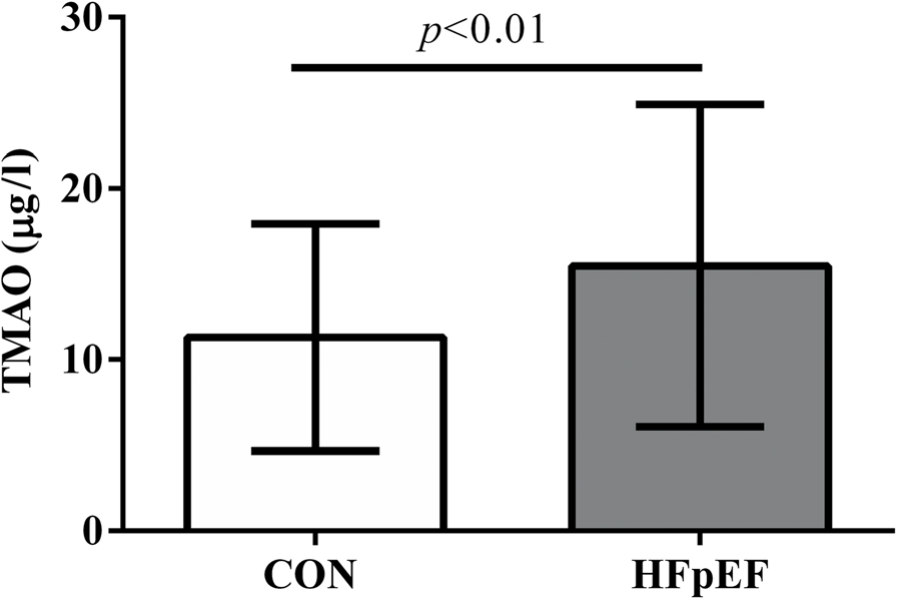
Comparison of plasma TMAO levels in CON and HFpEF groups. CON, control group. HFpEF, heart failure with preserved ejection fraction

Notably, plasma TMAO levels were significantly upregulated in HFpEF patients compared with healthy controls (12.65(9.32–18.66) μg/l vs 10.85(6.35–15.58) μg/l, *p* < 0.01) (Fig. 1).

### Comparison of clinical characteristics in different plasma betaine tertile groups and correlations of TMAO with eGFR

**Fig. 2:**
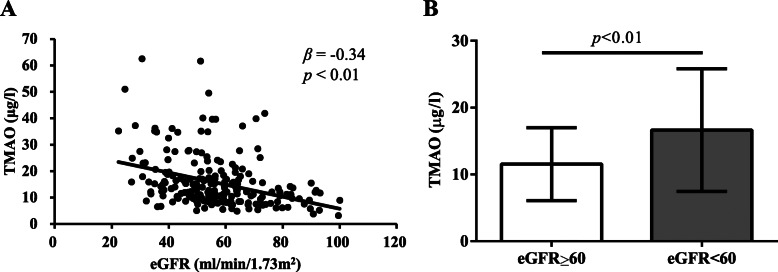
Correlation of plasma TMAO levels with eGFR. **a**, Spearman analysis of TMAO levels with eGFR. **b**, comparison of plasma TMAO levels between HFpEF patients with eGFR < 60 and eGFR ≥60

To investigate the changes of characteristics with TMAO levels, we further categorized the total study cohort into tertiles according to distribution of plasma TMAO. Subjects had lowest TMAO concentrations in tertile 1 (TMAO < 9.7 μg/l, *n* = 108), median TMAO concentrations in tertile 2 (TMAO = 9.7–15.58 μg/l, *n* = 109), and highest TMAO concentrations in tertile 3 (TMAO > 15.58 μg/l, *n* = 107). As shown in Table 2, there were more elderly subjects in higher TMAO tertile compared with lower TMAO tertile (62 ± 1 years in tertile 3 vs 61 ± 1 years in tertile 2 vs 58 ± 1 years in tertile 1, *p* < 0.01). Remarkably upregulated HbA1c% (6.1(5.9–6.5) in tertile 3 vs 6.1(5.7–6.1) in tertile 2 vs 5.7(5.5–6.1) in tertile 1, *p* = 0.03) and Cr (97(82.1–118.5) μmol/l vs 90.5(82.05–101.4) μmol/l vs 83.1(71.1–94) μmol/l, *p* < 0.01) levels were observed with increased TMAO concentrations. Accordingly, eGFR levels were decreased with increased TMAO tertiles (51.55(39.72–62.32) ml/min/1.73m^2^ in tertile 3 vs 56.22(49.27–65.64) ml/min/1.73m^2^ in tertile 2 vs 61.77(53.16–73.68) ml/min/1.73m^2^ tertile 1, *p* < 0.01). While no difference was found in other characteristics including sex, height, weight, BMI, SBP, DBP, HR, Glu, TG, TC, HDL, LDL, ALT, AST, WBC, CK-MB and EF% between different tertiles, it is noteworthy that the incidence of HFpEF significantly increased with increased TMAO tertiles (84 (78.5%) in tertile 3 vs 80 (73.39%) in tertile 2 vs 64 (59.26%) in tertile 1, *p* < 0.01). TMAO levels were inversely associated with eGFR score by Spearman analysis as shown in Fig. 2a (*β* = − 0.34, *p* < 0.01). Consequently, we further divided HFpEF patients into eGFR ≥60 group and eGFR < 60 group and observed significantly elevated TMAO concentrations in eGFR < 60 group compared to eGFR ≥60 group (14.18(10.4–23.06) μg/l vs 10.9(7.48–15.47) μg/l, *p* < 0.01, Fig. 2b).

**Table 2:** General characteristics of patients by tertiles of TMAO levels

	Tertiles of circulating TMAO			
	Tertile 1	Tertile 2	Tertile 3	*p*
	(< 9.7 μg/l) *n* = 108	(9.7–15.58 μg/l) *n* = 109	(> 15.58 μg/l) *n* = 107	
Male (n, %)	58 (53.7%)	70 (64.22)	72 (66.67)	0.1
Age (years)	58 ± 1	61 ± 1	62 ± 1	< 0.01
Height (cm)	162 ± 1	163 ± 1	164 ± 1	0.38
Weight (kg)	64 ± 1	67 ± 1	66 ± 1	0.36
BMI (kg/m^2^)	24 ± 0	25 ± 0	24 ± 0	0.77
SBP (mmHg)	131 ± 3	129 ± 2	133 ± 2	0.06
DBP (mmHg)	77 ± 1	76 ± 1	77 ± 1	0.57
HR (bpm)	72 ± 1	72 ± 1	72 ± 1	0.91
Glu (mmol/l)	5.2 (4.72–5.93)	5.34 (4.85–5.93)	5.42 (4.88–5.92)	0.38
HbA1c (%)	5.7 (5.5–6.1)	6.1 (5.7–6.1)	6.1 (5.9–6.5)	0.03
Cr (μmol/l)	83.1 (71.1–94)	90.5 (82.05–101.4)	97 (82.1–118.5)	< 0.01
eGFR (ml/min/1.73m^2^)	61.77 (53.16–73.68)	56.22 (49.27–65.64)	51.55 (39.72–62.32)	< 0.01
TG (mmol/l)	1.56 (1.06–2.18)	1.48 (0.98–2.09)	1.65 (1.36–2.73)	0.14
TC (mmol/l)	4.15 (3.64–5.48)	4.2 (3.57–5.18)	4.28 (3.65–5.23)	0.5
HDL (mmol/l)	1.19 (0.97–1.48)	1.1 (0.87–1.34)	1.01 (0.87–1.19)	0.05
LDL (mmol/l)	2.66 (2.04–3.42)	2.61 (2.25–3.24)	2.76 (2.22–3.45)	0.25
ALT (U/l)	21.7 (15.9–34)	22.5 (15.2–34.2)	24.2 (17.5–36.9)	0.6
AST (U/l)	22.2 (18.8–29.08)	22.7 (19.4–29.7)	23.5 (19–30.8)	0.51
WBC (10^9^/l)	6.4 (5.33–7.78)	6.6 (5.2–8)	6.5 (5.3–7.7)	0.3
CK-MB (U/l)	16.6 (12.1–21.9)	17.2 (12.1–25.45)	16.4 (13.1–22.6)	0.2
EF%	59 (57–65)	57 (54–64)	57 (55–64)	0.6
HFpEF (n, %)	64 (59.26%)	80 (73.39%)	84 (78.5%)	< 0.01

Continuous data are presented as mean ± SD or median (interquartile range), and categorical variables are presented as counts and percentage (%)

### Association and diagnostic value of TMAO levels with HFpEF and renal dysfunction

The association of plasma TMAO with HFpEF and renal dysfunction (eGFR < 60) were performed by logistic regression analysis (Table 3). Elevated TMAO levels were significantly and independently associated with the increased risk of HFpEF (OR = 3.49, 95% CI: 1.23–9.86, *p* = 0.02) after adjustment for other traditional risk factors including age, sex, SBP, HbA1c, Cr, TG, HDL and LDL. Not only that, TMAO was also an independent risk factor of renal dysfunction in HFpEF patients with an OR of 9.57 (95% CI: 2.11–43.34, *p* < 0.01) even after adjustment of age, sex, SBP, HbA1c, TG, HDL and LDL (Table 4).

**Table 3:** Association of TMAO levels with HFpEF

	OR	95% CI	*p*
Age	1.05	1.02–1.08	< 0.01
Sex	1.16	0.61–2.23	0.65
SBP	1.01	0.98–1.03	0.07
HbA1c%	1.33	1.05–1.69	0.02
Cr	1.01	0.95–1.03	0.15
TG	1.01	0.83–1.22	0.95
HDL	0.62	0.25–1.54	0.3
LDL	1.38	1–1.89	0.05
TMAO	3.49	1.23–9.86	0.02

Adjusted sex, age, SBP, HbAlc%, Cr, TG, HDL, LDL and TMAO for HFpEF

**Table 4:** Association of TMAO levels with renal dysfunction in HFpEF

	OR	95% CI	*p*
Age	1.04	1.01–1.08	0.02
Sex	7.15	3.49–14.65	< 0.01
SBP	1	0.98–1.01	0.6
HbA1c%	1.05	0.82–1.36	0.69
TG	0.93	0.77–1.12	0.42
HDL	1.05	0.38–2.9	0.92
LDL	1.21	0.86–1.71	0.26
TMAO	9.57	2.11–43.34	< 0.01

Adjusted sex, age, SBP, HbA1c%, TG, HDL, LDL and TMAO for renal dysfunction (eGFR < 60)

**Fig. 3:**
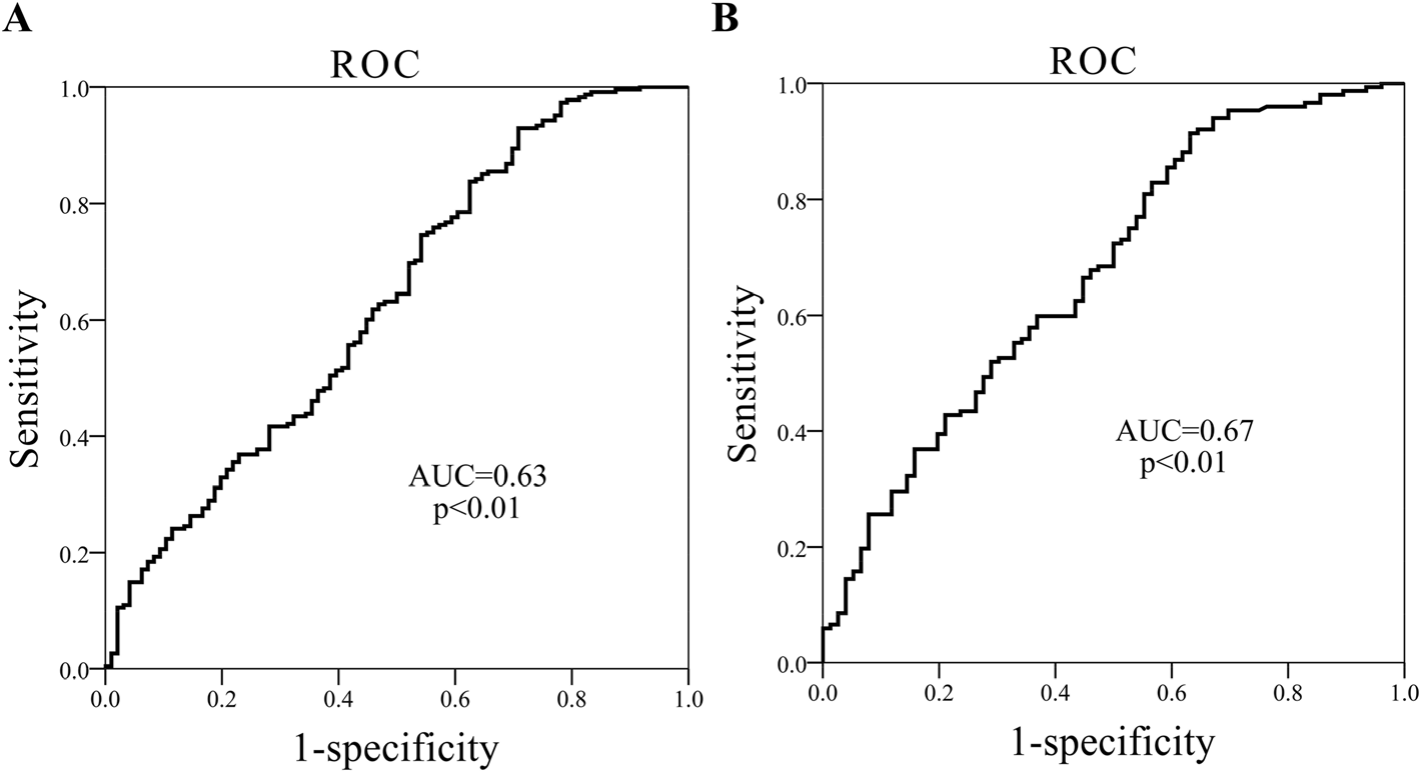
ROC curves of TMAO at discriminating HFpEF from CON, and impaired renal function from normal renal function. AUC indicates area under the receiver operating characteristic curve. **a**, ROC analysis of TMAO in discriminating HFpEF from CON, the likelihood of correctly predicting an event was up to 63% (AUC). **b**, ROC analysis of TMAO in discriminating renal dysfunction (eGFR < 60) from normal renal function (eGFR ≥60), the likelihood of correctly predicting an event was up to 67% (AUC)

At last, we performed receiver operating characteristic curve (ROC) analysis to explore the diagnostic role of TMAO in HFpEF and renal dysfunction. TMAO had good performance in discriminating HFpEF from healthy controls and discriminating eGFR < 60 from eGFR ≥60 in HFpEF patients, with AUCs of 0.63 (Fig. 3a) and 0.67 (Fig. 3b) (all, *p* < 0.01). The best cut-off threshold of TMAO in determining HFpEF and eGFR < 60 was 7.1 μg/l and 8.48 μg/l.

## Discussion

This is the first study to demonstrate that plasma TMAO levels are independently associated with both risk of HFpEF and renal dysfunction in HFpEF. Significantly elevated TMAO was observed in HFpEF patients compared to healthy controls and in patients with eGFR < 60 compared to patients with eGFR ≥60. Subjects with lower TMAO exhibited relatively higher eGFR and less frequency of HFpEF. In addition, inverse correlations were found between TMAO levels and eGFR. This study indicated that TMAO could be utilized as a noninvasive biomarker in diagnosis of HFpEF and renal function evaluation.

HFpEF is a complex disease involving an interplay of various factors. Mortality and morbidity rates in HFpEF population are amongst the highest for the world healthcare systems [13]. The Framingham Heart Study reported an annual mortality rate of 8.7% in HF patients with left ventricular ejection fraction > 50% and a fourfold higher mortality risk of those patients compared with healthy controls [14]. Patients of the HFpEF phenotype are often misdiagnosed and the severity of disease underestimated. So far, here are limited data to support use of disease specific therapy in HFpEF. Although intervention strategies and medicine based on traditional targets such as lipid metabolize pathway and angiotensin II (Ang II) have been developed and applied on the management of HFpEF, the growing prevalence of HFpEF still could not be abated due to lack of efficient biomarkers. Thus, a better understanding of the potential biomarkers and risk factors of HFpEF is critical for early prevention and proper management of the disease.

Growing evidences have indicated systemic inflammation as a key initiator which could introduce an extramyocardial origin in the progression of HFpEF [15]. Westermann and his colleagues reported an increase in the inflammatory cells CD3, CD11a and CD45 that secrete profibrotic growth factor TGF-β in HFpEF patients and suggested that inflammation might play a role in the diastolic dysfunction, which is the primary abnormality in HFpEF [16]. TMAO was demonstrated to enhance the expression of inflammatory genes in the aortic endothelium and smooth muscle cells and promote the adhesion of activated leukocytes to endothelial cells [17, 18]. Interestingly, in our study, we observed significantly elevated white blood cells as well as upregulated plasma TMAO in HFpEF group compared to healthy controls. These results suggested the potential role of TMAO in the pathogenesis of HFpEF by activation of immune cells and inducing endothelial inflammation. More than that, the incidences of HFpEF were increased with upregulated plasma TMAO levels in our study, which suggested a dose-dependent correlation of TMAO with HFpEF (Table 2). Consequently, results of the logistic analysis elucidated TMAO as an independent risk factor of HFpEF after adjustment for other traditional heart failure hazard factors such as age, sex, blood pressure, glucose and lipid in this study, the odds ratio of HFpEF would increase as high as 3.49 folds per 1og-1 μg/l TMAO increase. At last, we demonstrated the likelihood of TMAO in correctly predicting a HFpEF event was up to 63% (AUC = 0.63, *p* < 0.01, best cut-off = 7.1 μg/l, Fig. 3a). These results indicated that TMAO might be a useful biomarker for auxiliary diagnosis of HFpEF. However, further studies were needed for the verification of underlying mechanisms involving TMAO induced vascular inflammation in the onset of HFpEF.

Epidemiology studies have showed that patients with HFpEF were more likely to be older, have dyslipidemia and diabetes mellitus [19]. Although we failed to find any difference of CK-MB levels, a novel marker of myocardial injury, between HFpEF patients and controls, our study still showed worse lipid and glucose metabolic profiles in HFpEF group than their counterparts, as Glu, HbA1c, TG and LDL levels were significantly upregulated in HFpEF (Table 1). Notably, abnormal circulating TMAO concentrations were also reported to be involved with lipid and glucose homeostasis [20]. TMAO has been implicated in cholesterol transport and lead to accumulating of blood lipid [7]. Gao reported that dietary TMAO supplementation increased fasting insulin levels and exacerbated the impaired glucose tolerance in mice [21]. HbA1c is currently the gold standard for glucose monitoring and more closely related to the risk of chronic complications than random single or episodic glucose levels. Data from large epidemiological studies also suggested HbA1c as a potent predictor in cardiovascular disease [22]. We observed that HbA1c% levels were significantly elevated with increase in TMAO tertiles in our study cohort. Thus, we hypothesized that TMAO might also contribute to pathogenesis and progression of HFpEF through dampened lipid and glucose metabolic homeostasis. Yet, more studies are needed to verify the precise mechanisms of TMAO involved in lipid and glucose metabolism.

A cross-sectional study indicated that patients with heart failure with preserved ejection fraction (HFpEF) who had worse right ventricular free wall strain by echocardiography had worse estimated glomerular filtration rate (eGFR), and reduced eGFR was also associated with worse outcomes [23]. In ESCAPE (Evaluation Study of Congestive Heart Failure and Pulmonary Artery Catheterization Effectiveness) trial, up to 30% patients with acute HF was found to develop chronic kidney disease with significantly reduced GFR (< 60 ml/min/1.73 m^2^) [24]. Heart failure patients with impaired renal function had 1.28 folds increased risk of mortality than non-renal comorbidity patients [25]. There are several pathways in the setting of heart failure that contribute to a decline in the eGFR. Central venous congestion developed in HF was demonstrated to induce elevated interstitial pressure which subsequently resulted in decreased GFR [26]. In addition, long term treatment with diuretic may further exacerbate the renal function deterioration by reducing kidney blood flow [27]. However, HFpEF patients with renal comorbid conditions are often not screened for kidney disease until overt symptoms manifest, which is often too late in disease progression, due to nonspecific clinical symptoms and lack of efficient factor for disease stratification. Recent studies have suggested involvement of TMAO in declined renal function [28, 29]. After administration of radiolabeled TMAO to people, 94.5% of the labeled serum TMAO was excreted in urine within 24 h, which demonstrated that the kidney was the major organ for TMAO excretion in human and as well suggested the tight link of TMAO with renal glomerular filtration function [30]. Metabolomic analysis of urine samples from patients with chronic kidney disease (CKD) showed the remarkably elevated levels of TMAO compared with non-CKD subjects [31]. Coincidentally, we observed significantly higher TMAO in HFpEF patients with eGFR < 60 ml/min/1.73m^2^ (Fig. 2b) than those normal eGFR. Not only that, we revealed the remarkably decreased eGFR with increase in plasma TMAO as showed in Table 2 where lowest eGFR scores were found in subjects in highest TMAO tertile. Previous study has indicated that TMAO modestly correlated with eGFR and cystatin C, but did not correlate with high-sensitivity C-reactive protein 25,599,331. Similarly, Spearman analysis in our study also demonstrated the inverse correlation between TMAO levels and eGFR. These data all suggested the dose-dependent relationship of TMAO with impaired renal function. Accordingly, upregulated TMAO levels were showed to be independently associated with renal dysfunction (eGFR < 60 ml/min/1.73m^2^) after adjustment for sex, age, SBP, HbA1c, TG, HDL and LDL by logistic regression in the present study. In experimental mice models, Li demonstrated the contributory role of TMAO in the pathogenesis of endothelial dysfunction and CKD [32]. Here we expand those findings by revealing the risk predicting power of TMAO in HFpEF patients with impaired renal function compared to those with normal renal function (AUC = 0.67, *p* < 0.01, best cut-off = 8.48 μg/l, Fig. 3b). These results may suggest clinical utilities of TMAO for kidney function evaluation and risk stratification in HFpEF and also suggested possibility for new therapeutic approaches. It should be mentioned that diet and gut microbiota were closely linked with TMAO levels, while it was infeasible to unify those factors in the population of the present study. However, further investigations which involves well documented dietary and gut microbiota profile are warranted for better understanding of TMAO in disease progression and intervention.

### Limitations

This study has several limitations. First, this was a single center study, selection bias cannot be excluded. Second, as a cross-sectional study, we were not able to evaluate the future influence of TMAO on advance of HFpEF and its comorbidities. Additionally, we did not include other potential confounding factors such as patients’ nutritional status, recent diet, as well as microbiota profile, which might also influence the results.

## Conclusion

The present study explored the correlation of plasma TMAO with HFpEF and HFpEF with impaired renal function and revealed abnormal elevated TMAO with increased risk of HFpEF and worse renal comorbid conditions, and suggested the potential utility of plasma TMAO as a useful noninvasive biomarker for auxiliary diagnosis of HFpEF and renal function evaluation in HFpEF.

## Data Availability

The data and materials can be used with permission.

## Abbreviations

HFpEF: Heart failure with preserved ejection fraction
TMAO: Trimethylamine n-oxide
SBP: Systolic blood pressure
DBP: Diastolic blood pressure
BMI: Body mass index
HR: Heart rate
TC: Total cholesterol
TG: Triglyceride
HDL: High-density lipoprotein
LDL: Low-density lipoprotein
HbA1c: Glycosylated hemoglobin A1c
Glu: Fasting blood glucose
WBC: White blood cells
Cr: Creatinine
eGFR: Estimated glomerular filtration rate
ALT: Alanine aminotransferase
AST: Glutamic-pyruvic transaminase
CK-MB: Creatine kinase-MB
EF%: Percentage of ejection fraction
HPLC-MS: High performance liquid chromatography-tandem mass spectrometry
AUC: The area under the curve
ROC: Receiver-operating characteristic curve
